# Metal–Organic Frameworks for the Enhancement of Lithium‐Based Batteries: A Mini Review on Emerging Functional Designs

**DOI:** 10.1002/advs.202305280

**Published:** 2023-11-09

**Authors:** Anthony U. Mu, Guorui Cai, Zheng Chen

**Affiliations:** ^1^ Department of Nano and Chemical Engineering University of California San Diego 9500 Gilman Drive La Jolla CA 92093 USA; ^2^ Department of Chemical and Biomolecular Engineering University of Maryland 4418 Stadium Dr College Park MD 20742 USA; ^3^ Program of Materials Science and Engineering University of California San Diego 9500 Gilman Drive La Jolla CA 92093 USA; ^4^ Sustainable Power and Energy Center University of California, San Diego La Jolla CA 92093 USA

**Keywords:** cycling stability, lithium‐based batteries, metal–organic frameworks, wide operation voltage, wide‐temperature operation

## Abstract

Metal–organic frameworks (MOFs) have played a crucial role in recent advancements in developing lithium‐based battery electrolytes, electrodes, and separators. Although many MOF‐based battery components rely on their well‐defined porosity and controllable functionality, they also boast a myriad of other significant properties relevant to battery applications. In this mini‐review, the distinct advantages of MOFs in battery applications are discussed, including using MOFs to 1) scavenge impurities to increase cycling stability, 2) widen the operation temperature range of conventional electrolytes, 3) widen the operation voltage range of common electrolytes, and 4) employ as artificial solid‐electrolyte interphases to prevent lithium dendrite growth. Furthermore, subsisting challenges of developing these emerging MOF‐based battery technologies are discussed and guidance for shaping the future of this field is given.

## Graphical abstract

MOFs possess distinct advantages in scavenging impurities to increase cycling stability, increasing operation temperatures and voltages of common electrolytes, and can be used as artificial solid‐electrolyte interphases to prevent lithium dendrite growth. In this mini‐review, the emerging battery applications of metal–organic frameworks (MOFs) are outlined, and a perspective of future MOF‐based battery technologies is proposed.

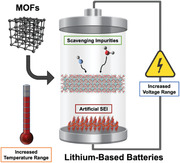

## 1 Introduction

As the global population continues to grow and modern technology advances, the demand for renewable energy and energy storage technologies increases. The growth of both portable electronics and electronic vehicle markets necessitates the development of energy storage with longer cycling life, more reliable safety, and higher energy density than today's technologies.^[^ ^1^ ^]^ Moreover, the development of environmentally friendly renewable energy technologies calls for reliable electrochemical storage devices.^[^ ^2^ ^]^ In this regard, lithium‐ion batteries (LIBs) have seen a meteoric rise in popularity due to their high theoretical energy densities. Despite considerable efforts invested in this field, state‐of‐the‐art battery systems are approaching the threshold of their performance limits.^[^ ^3^ ^]^ To overcome these challenges, lithium–metal batteries (LMBs) demonstrate even higher capacities and have recently been referred to as the “holy grail” for next‐generation energy systems.^[^ ^4^, ^5^, ^6^, ^7^ ^]^

Currently, both LIBs and LMBs continue to face countless restrictions to their electrochemical performances, notably, due to their capacity fading during repeated electrochemical cycling.^[^ ^8^ ^]^ A major factor of this loss is due to corrosive hydrogen fluoride (HF) that is generated through the hydrolysis of electrolytes.^[^ ^9^, ^10^ ^]^ Furthermore, these effects are exacerbated in complex operating environments due to exposure to high voltages^[^ ^11^ ^]^ and elevated temperatures.^[^ ^12^, ^13^ ^]^ Lithium dendrite growth also remains a recurring problem for lithium‐based batteries, leading to safety hazards and short lifespans.^[^ ^14^ ^]^ Although a significant amount of research exists in this field, many of these challenges persist. Drawbacks in these current systems drive investigation into the development of new materials for energy storage devices.

Metal–organic frameworks (MOFs) are a class of porous crystalline materials comprised of metal‐based nodes coordinated to organic ligands that have attracted wide research interests in recent years.^[^ ^15^, ^16^, ^17^ ^]^ Due to their modular nature, the combinations of possible metallic elements and organic linkers are boundless, leading to the experimental synthesis of over 90 000 MOFs thus far.^[^ ^18^ ^]^ Before their introduction into the battery field, MOFs have experienced great success in various applications, such as gas adsorbents,^[^ ^19^ ^]^ sensors,^[^ ^20^ ^]^ drug carriers,^[^ ^21^ ^]^ and catalysts,^[^ ^22^ ^]^ owing to their extensive chemical functionality and topological control. Recent efforts have pushed MOFs into the field of energy materials due to their inherent porosity, well‐ordered networks, and chemical stability, which allow for ion compatibility and electrochemical robustness.^[^ ^23^ ^]^ Consequently, electrode materials^[^ ^24^ ^]^ and separator components^[^ ^25^ ^]^ have benefited from the properties of MOFs. Furthermore, MOF‐based electrolytes have achieved ionic conductivities of up to 10^−3^ S cm^−1^, demonstrating promising results for next‐generation battery applications.^[^ ^26^, ^27^, ^28^ ^]^ The recognized mechanism of these MOF‐based electrolytes, electrodes, and separators relies on well‐defined pore architectures of MOFs that can facilitate fast ion transport, host active materials, and selectively screen ions, respectively. While this exploits one defining trait of MOFs, they exhibit countless other important properties for battery applications, such as large hygroscopic adsorption capacities, high thermal stabilities, excellent electrochemical stabilities, and high mechanical robustness. These properties have recently been used to rectify the aforementioned challenges in current battery materials.^[^ ^29^ ^]^

**Figure 1:**
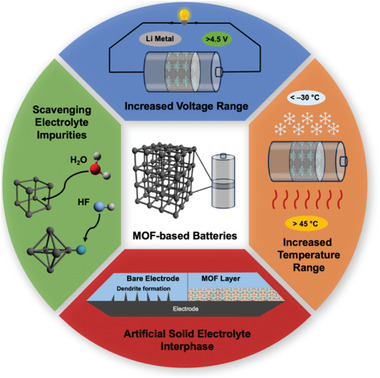
Schematic showing the emerging battery applications of MOFs.

In this mini review, we detail these emerging MOF applications in batteries used to 1) scavenge impurities to improve cycling stability, 2) widen the operating temperature range of electrolytes, 3) widen the operating voltage range of electrolytes, 4) and operate as artificial solid‐electrolyte interphases (SEIs) to prevent lithium‐dendrite formation (**Figure** **1**). The unique advantages of each MOF material are comprehensively discussed and current challenges are summarized. Lastly, the prospective solutions for the future fabrication of MOF‐based batteries are presented.

## 2 MOFs Used as Scavengers to Improve Cycling Stability

**Figure 2:**
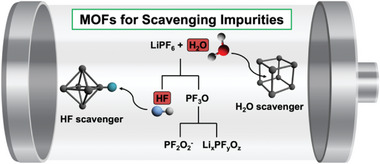
Proposed reaction pathways for the formation of common impurities in lithium batteries and relative scavenging processes by MOFs.

Transition metal dissolution and electrode erosion are the well‐known origins of capacity decay and the limited stability of LIBs during repeated cycling.^[^ ^30^ ^]^ Conventional lithium salts, such as LiPF_6_, readily undergo hydrolysis reactions with trace water, potentially leading to the generation of highly corrosive HF (**Figure** **2**), which acts as a main factor in the aforementioned detrimental phenomena. Furthermore, gaseous product formation through hydrogen (or hydrocarbon molecules) and oxygen evolution reactions also leads to cell swelling and other safety concerns. These destructive processes are intensified at higher operating temperatures, preventing the development of safer and longer lifespan LIBs and LMBs. Although attempts to ameliorate these effects largely rely on coating the cathode to protect against erosion,^[^ ^31^ ^]^ the other adverse effects of HF generation fail to be addressed by this method. In this regard, various additives with high affinities for water and HF have been reported to scavenge these harmful impurities.^[^ ^32^ ^]^

**Figure 3:**
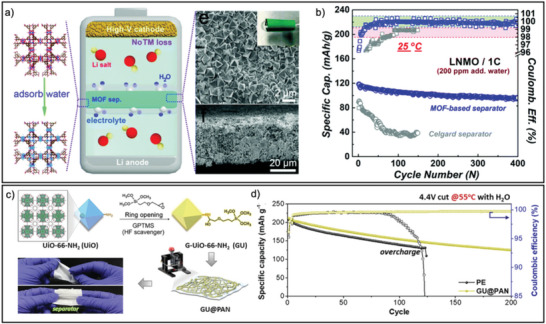
a) Schematic showing a HKUST‐1 MOF‐based membrane as a water scavenger. b) Cycling performances of Celgard separator and MOF‐based in‐built water scavenger in Li||Ni_0.5_Mn_1.5_O_4_ cells at 25 °C (1 C current rate). c) Illustration of scavenging moiety functionalized MOFs and related membrane fabrication process for scavenging HF leading to d) improved cycling stability even with 500 ppm water at 55 °C. (a,b) Reproduced with permission.^[^ ^33^ ^]^ Copyright 2020, Royal Society of Chemistry. (c,d) Reproduced with permission.^[^ ^34^ ^]^ Copyright 2023, Wiley‐VCH.

MOF materials comprised of abundant absorption sites and porous crystalline structures demonstrate promising abilities to scavenge impurities, facilitating HF, water, and gas capture. For example, the tenacious water‐scavenging properties of a copper‐based MOF (HKUST‐1) were employed in fabricating a battery separator for LIBs.^[^ ^33^ ^]^ As a result, the removal of moisture has led to a decrease in the hydrolysis of electrolytes, granting an increased capacity retention of 72% after 400 cycles in Li||LiNi_0.8_Co_0.1_Mn_0.1_O_2_ cells, even in the presence of an additional 200 ppm of water in the electrolyte (**Figure** **3**a,b). Even after increasing the electrolyte water content to 800 ppm, acceptable performance was sustained. The advantages of MOF materials are not limited to their inherent impurity scavenging properties, as they also possess ease of functionalization, providing more options to install additional scavenging moieties.

By equipping an amine‐functionalized zirconium‐based MOF (UiO‐66‐NH_2_) backbone with (3‐glycidyloxypropyl) trimethoxy‐silane (GPTMS) as an HF scavenger to form GPTMS‐functionalized UiO‐66‐NH_2_, scavenging abilities were extended beyond water to include HF.^[^ ^34^ ^]^ The resulting all‐impurity scavenging separator was constructed from the combination of functionalized MOFs and polyacrylonitrile binders, which demonstrated the effective removal of harmful HF. In addition, the CO and CO_2_ confinement capabilities of UiO‐66‐NH_2_ were important in lessening the gaseous volume expansion leading to cell swelling due to gas generation, thereby the deterioration of battery components was decreased (Figure 3c,d). The scavenging of these major impurities led to an improved capacity retention of 75% after 200 cycles, performing even at higher temperatures (55 °C) and demonstrated stability even with an additional 500 ppm of water in the electrolyte.

The effective moisture, HF, and gas adsorbing abilities of the presented MOFs demonstrate a promising method for the enhancement of LIB cycling performances. Considering that these impurities are major factors in transition metal dissolution and electrode erosion, the introduction of the aforementioned MOF separators led to an enhanced capacity retention of LIBs, even with a significant amount of water in the electrolyte during cell assembly. The current investigations showcase the possibilities of utilizing MOF separators to mitigate the deleterious effects of common electrolyte impurities.

## 3 MOFs Used to Widen the Operation Temperature Range of Electrolytes

**Figure 4:**
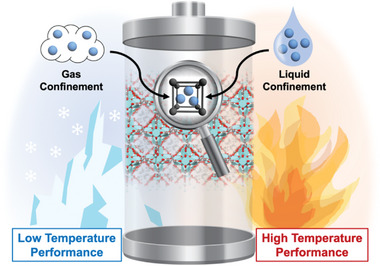
Schematic showing the MOF‐based electrolytes for batteries operating under extreme temperatures.

Generally, solid‐state electrolytes possess many inherent advantages to their liquid counterparts in terms of thermal stability and eliminating the use of flammable organic solvents. Despite their improved safety, solid‐state electrolytes tend to exhibit inferior ionic conductivity and poor mechanical properties. To circumvent the drawback of both liquid and solid‐state electrolytes, quasi‐solid electrolytes can be expected to enhance ionic conductivities while reducing the amounts of dangerous organic solvents. As quasi‐solid electrolytes are prepared by hosting a minuscule amount of liquid or gas electrolyte inside a porous matrix, physical and chemical properties can be tuned to enable a much wider range of safe operating temperatures, enabling both high and low‐temperature applications (**Figure** **4**).

**Figure 5:**
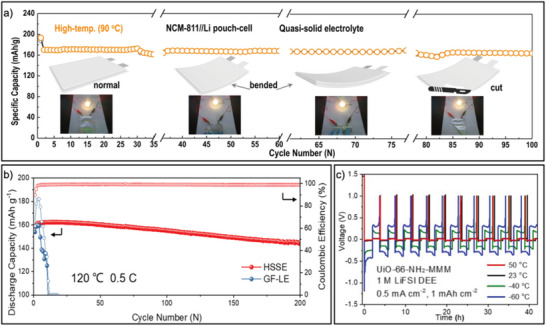
a) Cycling performance of LMB pouch cell assembled with MOF‐based quasi‐solid electrolyte at 90 °C and after sustaining damage. b) Cycling performance of hybrid solid‐state electrolyte with activated Al‐based MOFs in comparison with previously reported liquid electrolytes. c) Wide‐temperature testing of Li||Cu with UiO‐66‐NH_2_ mixed‐matrix membrane‐trapped electrolyte systems. (a) Reproduced with permission.^[^ ^35^ ^]^ Copyright 2023, Springer Nature. (b) Reproduced with permission.^[^ ^36^ ^]^ Copyright 2023, Royal Society of Chemistry. (c) Reproduced with permission.^[^ ^37^ ^]^ Copyright 2022, American Chemical Society.

Nanoconfinement and sub‐nanoconfinement of liquids have been shown to substantially change their physicochemical properties, such as boiling points, melting points, ion transport abilities, etc. Using these effects, CuBTC MOF was functionalized with poly(sodium 4‐styrenesulfonate) and employed as a host material to confine the liquid electrolyte, bis(trifluoromethanesulfonyl)imide (LiTFSI) in propylene carbonate (PC).^[^ ^35^ ^]^ Although the conventional electrolyte suffered from low decomposition temperatures at ≈100 °C, an increase in decomposition temperature of up to 200 °C was observed after fabrication of the quasi‐solid electrolyte. The resulting LMB pouch cells containing this MOF‐based quasi‐solid electrolyte demonstrated 89% capacity retention after 200 cycles at high working temperatures of 90 °C, even functioning after being bent and cut (**Figure** **5**a). Similarly, further enhancement of LMB operating temperatures was achieved through the MOF‐facilitated (HKUST‐1) confinement of a Li salt‐containing ionic liquid ([EMIM][TFSI]).^[^ ^38^ ^]^ The high thermal stability of ionic liquids in contrast to conventional electrolyte solvents was exploited through the immobilization in MOF nanopores. Consequently, the resulting quasi‐solid electrolyte was employed in LiFePO_4_||Li batteries, achieving a 92% capacity retention after 100 cycles at 0.5 C.

In addition to manipulating the boiling points of confined solvents within sub‐nanoscale environments, MOFs themselves have also been manipulated to widen the operating temperatures of these electrolytes. For instance, to further increase the high‐temperature stability of a MOF‐based solid‐state electrolyte in LMBs, an aluminum‐based MOF (MIL‐96) was selected due to the high binding energy of Al─O bonds and exposed Al^3+^ coordination sites upon activation, which guaranteed excellent stability of microporous ion channels at high temperatures and enhanced Li^+^ conductivity (Figure 5b).^[^ ^36^ ^]^ A hybrid solid‐state electrolyte was prepared with the aluminum‐based MOF and polyvinylidene fluoride (PVDF), leading to exceptional stability compared to its liquid electrolyte counterparts. After 200 cycles at 120 °C, the LiFePO_4_||Li batteries exhibited 93% capacity retention and maintained 99% Coulombic efficiency. Furthermore, these results were in direct contrast of the copper‐containing MOF (Cu‐MOF), which could not cycle at such high temperatures due to its poor stability.

**Figure 6:**
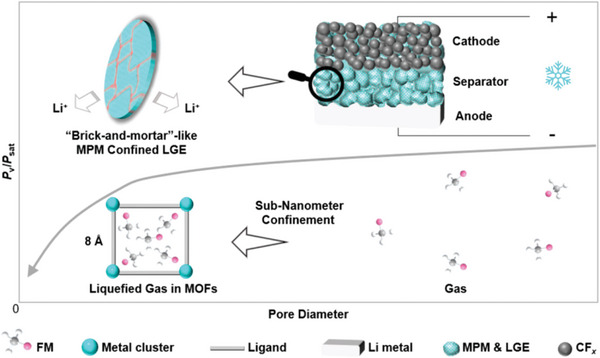
Schematic showing the mechanism of nano‐confinement effects for lowering the equilibrium pressure of liquefied gas and the implementation of MPM‐based liquefied gas electrolytes (LGE) for Li batteries. Reproduced with permission.^[^ ^39^ ^]^ Copyright 2021, Springer Nature.

While improving the operating temperature range of batteries is crucial for long‐term stability, batteries that can function in low temperatures are equally necessary for applications in extremely cold conditions, such as in outer space or deep ocean exploration.^[^ ^40^ ^]^ In this context, Cai et al. utilized MOF‐confinement effects of gaseous molecules (fluoromethane) in sub‐nanometer MOF pores to exploit the capillary condensation effect (**Figure** **6**).^[^ ^39^ ^]^ The fluoromethane‐loaded MOF‐polymer membranes formed a liquefied gas electrolyte that was able to operate below its vapor pressure. Furthermore, the resulting Li||CF*_x_* cells demonstrated significantly higher capacities (≈500 mAh g^−1^) at −40 °C than those with conventional Celgard membranes (<0.03 mAh g^−1^). To reduce the strong friction between the nanopore walls and confined electrolyte molecules without sacrificing the strong confinement effect, the same research group systematically adjusted the microenvironments of nanopore chemistry by modifying the linker group chemistry of the UiO‐66 series.^[^ ^37^ ^]^ These functionalized MOFs were shown to have enhanced trapping capabilities for volatile low‐temperature electrolyte solvents (Figure 5c). This improvement not only addresses safety concerns but also allows a wider range of working temperatures. The study also revealed aggregated solvation structures, modulated solvent molecular configurations, and tunable transport mechanisms from quasi‐solid to quasi‐liquid in functionalized MOFs, which deviated from the bulk counterparts.

These quasi‐solid electrolytes prepared by hosting liquid and gas electrolytes in porous MOF matrices circumvent many of the shortcomings of both liquid electrolytes and solid‐state electrolytes. The capillary condensation and confinement effects of liquid and gas electrolytes not only alter their physicochemical properties, but also maintain their ionic conductivities, highlighting the potential of these quasi‐solid electrolytes in various extreme temperature battery applications. While the examples demonstrate that high operating temperatures were achieved through the confinement of conventional liquid and ionic liquid electrolytes and further enhancing the thermal stability of the MOF itself, low operating temperatures were also achieved by confining gaseous electrolyte molecules. The results underscore the high potential of MOF‐confined electrolytes as a novel approach for the development of next‐generation batteries.

## 4 MOFs Used to Widen the Operation Voltage Range of Electrolytes

**Figure 7:**
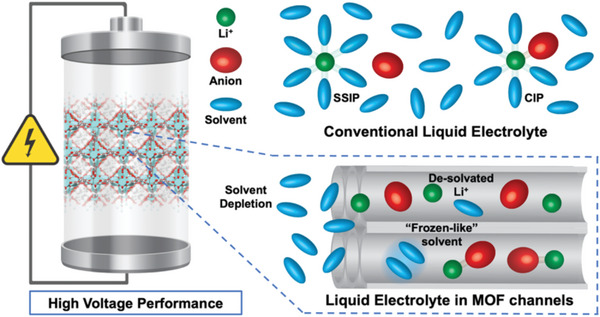
Schematic showing the MOF‐based electrolytes used to increase the operating voltage range of batteries.

Pairing lithium metal with various high‐voltage cathode materials holds great promise in the pursuit of high‐energy‐density batteries. However, this approach poses demanding requirements for electrolytes, necessitating both excellent oxidative stability and high reversibility toward lithium metal. At high voltages, the oxidation of solvent molecules restricts the electrochemical stability window of the electrolytes. Adding more salts into typical diluent electrolytes to form high‐concentration electrolytes can eliminate free solvents. It also leads to a shift in solvation structure from the solvent‐separated ion pair‐dominated configuration to the contact‐ion pair‐dominated configuration, resulting in denser SEI enriched with anion‐derived inorganic species (**Figure** **7**). Consequently, high‐concentration electrolytes exhibit aggregated solvation structures with reduced free solvent content, thereby simultaneously enhancing Li metal compatibility and oxidative stability.

Despite the promising benefits, the practical implementation of this approach is hindered by the high cost and viscosity of the salts, making them unsuitable for real‐world battery cells. An alternative strategy involves introducing a diluent solvent to create locally high‐concentration electrolytes, reducing viscosity without noticeably compromising the solvation structures. Nonetheless, this dilution process results in mediocre ionic conductivity, leading to inferior rate performances and even causing short circuit events in high‐loading cells. Furthermore, even concentrated electrolytes are not completely immune to solvent‐related decomposition issues, because free solvent molecules can still form during the desolvation of Li^+^ ions on the electrolyte surface. While solid‐state electrolytes offer a solution to these problems by entirely avoiding solvent decomposition, their low ionic conductivity still remains as a challenge to satisfy the demand for practical batteries. Consequently, liquid electrolytes remain the most widely used option for lithium‐ion batteries.

**Figure 8:**
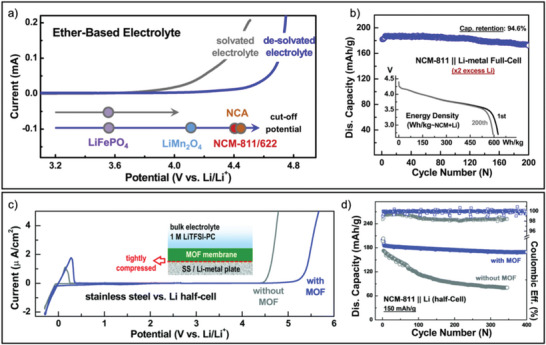
a) Improved oxidative stability of the MOF‐based Li^+^ desolvated electrolyte and the b) discharge capacity against cycle number collected from NCM‐811||Li‐metal full‐cells using this electrolyte. The inset shows the corresponding galvanostatic discharge curves versus the gravimetric energy density. c) Linear sweep voltammetry curves of typical electrolyte and the prepared solvent‐depleted electrolyte in MOFs. The inset schematically illustrates the cell configuration for the measurement. d) Cycling performance of the NCM‐811||Li half‐cell using the MOF‐based electrolyte. (a,b) Reproduced with permission.^[^ ^41^ ^]^ Copyright 2020, Elsevier. (c,d) Reproduced with permission.^[^ ^42^ ^]^ Copyright 2020, Royal Society of Chemistry.

Desolvated Li^+^ ions in MOF (ZIF‐7) pores were discovered in ether‐based electrolytes, where the ether solvents exhibited a “frozen” behavior within the MOF pores (**Figure** **8**a,b).^[^ ^41^ ^]^ This “frozen‐like” solvent, combined with the crystal‐like salt solute in MOFs, enabled the ether‐based electrolytes to operate stably at over 4.5 V. Based on the aforementioned mechanism, MOFs (ZIF‐71) with narrow pore sizes (4.2 Å) were used as a unique electrolyte solvation sheath filter to isolate free or weakly‐coordinated solvents from electrode contact. As a result, these special electrolytes composed of only strongly coordinated solvent molecules were achieved. This MOF‐filtered electrolyte, containing LiTFSI in PC, exhibited remarkably widened electrochemical stability windows of up to 5.2 V. Similarly, to further deplete solvents contained in electrolytes, which is considered an effective method for improving the electrochemical performances of high‐energy‐density LMBs, a more aggregative electrolyte configuration than a saturated liquid electrolyte was constructed in MOFs (HKUST‐1).^[^ ^42^ ^]^ The solvated Li^+^ ions in an ester‐based solvent encounter a partial desolvation process for the adaptation of narrow pore spaces. This unique electrolyte configuration allowed for significant expansion of the electrochemical oxidation stability window for MOF‐confined LiTFSI salt in the PC solvent system, increasing it from the original 4.5 to 5.4 V, while also exhibiting superior Li metal compatibility (Figure 8c,d). In addition, the suppression of lithium reactivity with electrolytes was also achieved through a MOF‐based nanoporous separator.^[^ ^43^ ^]^ The small nanopores of the separator partially desolvated Li^+^ ions and created a confined environment that deactivated solvents for electrochemical reduction before Li metal deposition.

**Figure 9:**
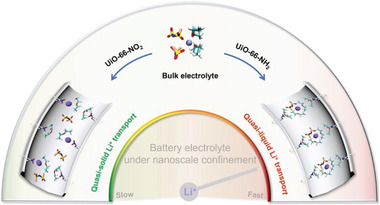
Schematic showing the ultrafast Li^+^ ion migration and tunable transport mechanism inside MOFs. Reproduced with permission.^[^ ^37^ ^]^ Copyright 2022, American Chemical Society.

In summary, the quasi‐solid electrolyte formed by the MOF‐confined lean liquid electrolytes offers a promising combination of the advantages found in both conventional liquid electrolytes and solid‐state electrolytes. The MOFs act as a solid shell, preventing direct contact between the solvent and electrodes, while also deactivating the free solvent, depleting solvated Li^+^ ion, and even promoting electrolyte aggregation (Figure 7). Differing from solid electrolytes, confined liquids in MOF pores allow for high Li^+^ conductivity. A quasi‐liquid transport mechanism was found in UiO‐66‐NH_2_ confined nanoporous spaces, which proved useful for addressing stability concerns associated with volatile organic electrolytes while simultaneously endowing ultrafast transport of solvates (**Figure** **9**).^[^ ^37^ ^]^ Similarly, a liquid‐electrolyte‐like transport mechanism was reported in the MOF‐confined LiPF_6_ in PC‐contained open metal sites that effectively trapped PF_6_^−^ anions, thus hindering their mobility.^[^ ^44^ ^]^ Furthermore, the fixed counter‐ions on the MOF channels generate a negatively charged field, facilitating the transport of Li^+^ ions and increasing the effective Li^+^ ion transfer number compared to standard liquid electrolytes. These findings highlight the potential of MOF‐confined electrolytes as an innovative approach to enhance the performance of advanced battery systems.

## 5 MOFs Used as Artificial Solid‐Electrolyte Interphases

The in situ formation of SEI interfacial films on the Li metal surface, utilizing electrolyte additives and other methods, has proven to be an effective strategy for passivating the Li metal and preventing parasitic electrolyte decomposition. However, the unsatisfactory mechanical robustness of the vulnerable SEI struggles to effectively mitigate volume expansion. Furthermore, the low modulus of the as‐formed SEI layers commonly cannot withstand the mechanical deformation induced by dendrite growth. As for artificial SEI layers, integrating inorganic Li^+^ conductors pose difficulties due to their brittle nature. In contrast, polymeric Li^+^ conductors can form conformal coatings on Li metal with better integrity. Nevertheless, artificial SEI layers composed of polymers typically encounter poor conductivity issues, impacting the rate performance of lithium metal batteries.

**Figure 10:**
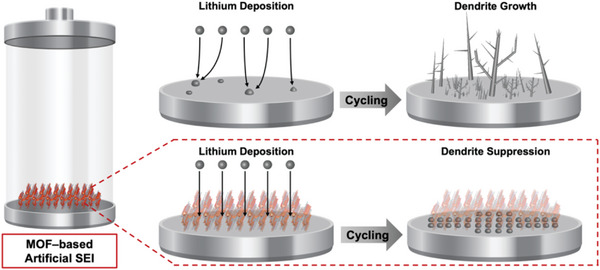
Illustration of comparison between dendrite formation on the bare electrode and dendrite suppression with MOF‐based artificial SEI layer.

**Figure 11:**
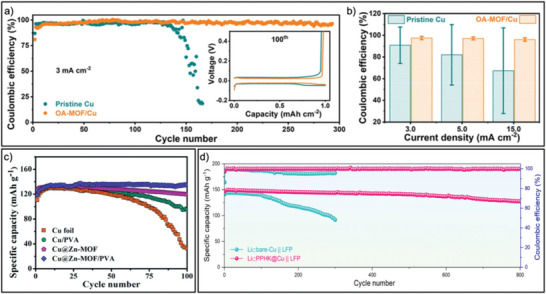
a) Evaluation of Coulombic efficiency plots of Li||Cu cells based on pristine Cu and the OA‐MOF/Cu at 3 mA cm^−2^ with a constant lithiation capacity of 1 mAh cm^−2^. Inset: corresponding galvanostatic Li plating/stripping curves at the 100th cycle. b) Average Coulombic efficiency and corresponding standard deviation for Li||Cu cells based on pristine Cu and OA‐MOF/Cu at 3, 5, and 15 mA cm^−2^. c) Cycle performance of Li||LiFePO_4_ full cells made with the artificial SEI protected Cu foil at 1 C. d) Long‐term cycling stability of the PHK@Cu versus bare‐Cu cells tested at 1 C. (a,b) Reproduced with permission.^[^ ^45^ ^]^ Copyright 2021, Wiley‐VCH. (c) Reproduced with permission.^[^ ^46^ ^]^ Copyright 2019, Royal Society of Chemistry. (d) Reproduced with permission.^[^ ^47^ ^]^ Copyright 2022, Wiley‐VCH.

MOF materials hold great promise as efficient artificial SEI layers, offering both mechanical strength and ionic conductivity. The chemical environment of MOF pores can be engineered to manipulate the Li^+^ ion transport behavior. The high mechanical strength of MOF particles poses a high potential for MOF films as artificial SEI layers to avoid Li dendrite puncturing (**Figure** **10**), although films assembled from isotropically grown bulk MOF nanoparticles are mechanically brittle. In this context, 2D MOF nanosheets with flexible skeletons offer remarkable advantages compared to bulk MOF grains. An open‐architecture MOF film constructed by vertically growing 2D MOF nanosheets presents stereoscopic lithiophilic sites.^[^ ^45^ ^]^ This unique configuration serves as a dynamic solid‐electrolyte interphase (SEI), exhibiting elastic expansion and contraction of the volume of stereoscopic lithiophilic sites. The self‐adjustment distribution of lithiophilic sites of the vertical growth of MOF nanosheets enables the homogenization of Li^+^ ion flux, smart control of Li mass transport, and compact Li deposition, which leads to improved cycling performance (**Figure** **11**a,b). These findings demonstrate the significant potential of 2D MOF nanosheets in advancing lithium‐ion battery technology through the efficient manipulation of lithiophilic sites and the formation of dynamic SEI.

Incorporating polymer binders as a “glue” to cement rigid MOFs has also been proven as an alternative strategy to address the inherent brittleness of bulk MOF assemblies. For instance, a MOF/polymer composite SEI layer was formed by in situ growth of a Zn‐MOF on Cu foil and spin‐coating polyvinyl alcohol solution to enhance flexibility.^[^ ^46^ ^]^ In addition, the polar O─H and Zn─N bonds in MOFs contributed to excellent electrolyte wettability and high Li^+^ ion flux, reducing surface concentration gradients. Nanoporous expanses within MOFs effectively screen ions and hinder anion migration, leading to enhanced Li^+^ ion migration with uniform Li^+^ ion flux and inhibition of Li dendrite formation. The artificial MOF‐polymer composite SEI film efficiently adapts to the changes in volume during the cycle and also exhibits a decent ionic conductivity, thereby significantly extending battery cycle life (Figure 11c). Furthermore, a polymer with abundant lithiophilic sites was introduced into MOFs to mitigate the impedance between MOF grain boundaries.^[^ ^47^ ^]^ The polymer then acts as a “chain” that interlinks Li “blocks” stored within the MOF pores. The MOF pores effectively compartmentalize bulk Li deposition, creating a 3D matrix for Li storage, which results in low‐barrier and dendrite‐free Li plating/stripping with exceptional Coulombic efficiency (Figure 11d). Additionally, the N‐rich polypyrrole component guides rapid Li^+^ infiltration/extrusion and serves as the nucleation site for isotropic Li growth.

The functions of MOF‐based artificial films are not limited to condensed and robust SEI films. In fact, they can also serve as electrolyte modulators and ion‐transport rectifiers on electrodes.^[^ ^44^, ^48^ ^]^ By dispersing them in the electrolyte or by drop‐casting on the electrodes, the MOF layer undergoes a transformation into an ion‐conducting interphase, facilitating preferential Li^+^ ion transport when the liquid electrolyte is introduced during battery assembly. The confined electrolyte within the MOF pore channels induces partial desolvation of Li^+^ ions, effectively lowering the activation energy for charge transfer during Li deposition. Furthermore, the strong binding of anions to the MOFs results in increased lithium transport numbers and effectively suppresses the formation of ion‐concentration gradients in full cells. These insights into the behavior of MOF‐coated electrodes shed light on the potential for designing effective SEI layers and improving the performance and safety of lithium‐ion batteries. Further investigation into the intricate interactions between MOFs and Li^+^ ions will advance the understanding of battery interfaces and guide the development of next‐generation energy storage technologies.

## 6 Conclusions and Outlook

In recent times, increasing attention has been on MOF‐based materials for battery applications. Beyond their well‐defined porosity, MOF‐based materials also exhibit large hygroscopic adsorption capacities, high thermal stabilities, excellent electrochemical stabilities, and mechanical robustness, which offer solutions to the current challenges in both LIBs and LMBs, including the hydrolysis of electrolytes, mediocre thermal and electrochemical stabilities, and poor compatibility of common electrolytes with electrodes. As detailed in this mini‐review, the properties of MOF‐based materials were in remarkable alignment for each of these challenges. Nevertheless, the exploration of novel applications using MOF‐based materials remains in its nascent stages and several challenges and issues need to be addressed. Some of the key problems facing this field are as follows:

1. Despite the extraordinary impurity scavenging abilities of the MOF‐based separators and additives, there exists a finite adsorption capacity to the MOF materials. For this reason, using similar functionalization strategies to install scavenging moieties on other MOF‐based battery components, such as electrolytes, is important to broaden the capabilities of this field.
2. Although the effects of nanoconfinement on liquid/gas electrolytes toward their melting/boiling points are well‐known, the fine‐tuning of pore size in comparison to confined molecules has not been thoroughly studied. In order to attain the threshold temperature range for MOF‐based electrolytes, a fundamental investigation should be done to give us insight into optimal electrolyte/MOF combinations.
3. As the limiting factor of electrochemical stability is often the solvent molecules, much of the focus is toward solvent depletion to improve oxidative stability. In this context, fully solid‐state MOF‐based electrolytes have the advantage since they are only comprised of highly electrochemically stable MOFs and lithium salts. While these systems typically lack adequately high ionic conductivities, this represents another important area for research. Increased focus on developing solvent‐free MOF‐based electrolytes will be crucial for reaching the limit of electrochemical stability.
4. While examples of the MOF‐based SEI films demonstrate effective Li dendrite suppression, there is a lack of focus on the mechanical strength of these layers. In order to draw reliable structure‐property relationships between certain MOF materials and inhibition of dendrite growth, mechanical properties (bulk modulus, shear modulus, etc.) are expected to be investigated further.
5. Preparation of these MOF‐based batteries requires costly materials, such as zirconium and ionic liquids. Moreover, the synthesis and processing of MOFs are made even more complex with the introduction of grafted scavengers and polymers. Utilization of these MOF materials requires significant effort and resources, which is a major obstacle in the commercialization of these batteries. Thus, the selected MOFs should maintain a balance between their synthetic cost and desirable properties and maintain an emphasis on using inexpensive starting materials as well as fewer synthetic steps.
6. MOFs possess many more unexplored characteristics that could prove valuable for battery materials. For example, while the majority of MOFs are insulating materials, recent strategies have been employed to develop electrically conductive MOFs,^[^ ^49^ ^]^ further permitting their use for cathode materials in energy storage devices.^[^ ^50^, ^51^, ^52^ ^]^ Moreover, the properties of MOFs discussed in this review have not been considered for other types of batteries, such as in other metal‐based batteries (Zn, Na, Al, etc.), and will most likely provide the same benefits as they have shown toward Li‐based batteries.

The development and research of MOF‐based battery materials based on their less widely utilized attributes is still in its infancy but has proven to be important in the future development of LIBs and LMBs. The underlying mechanisms for enhancing the performance of these materials remain uncertain, necessitating further research to achieve a comprehensive understanding. Significant progress is needed before these materials can be effectively employed in real‐world batteries. As greater attention is directed toward exploring these advantages of MOFs, the challenges presented in this mini‐review will be addressed with more systematic studies, leading to the realization of the potential of MOFs in battery applications.

## Conflict of Interest

The authors declare no conflict of interest.
